# *In vitro *inhibition of monkeypox virus production and spread by Interferon-β

**DOI:** 10.1186/1743-422X-9-5

**Published:** 2012-01-06

**Authors:** Sara C Johnston, Kenny L Lin, John H Connor, Gordon Ruthel, Arthur Goff, Lisa E Hensley

**Affiliations:** 1 Virology Division, United States Army Medical Research Institute of Infectious Diseases, 1425 Porter St. Fort Detrick, Frederick, MD 21702, USA; 2 Toxicology Division, United States Army Medical Research Institute of Infectious Diseases, 1425 Porter St. Fort Detrick, Frederick, MD 21702, USA; 3 Microbiology Department, Boston University School of Medicine and Microbiology, 72 E.Concord St R-Bd Boston, Boston, MA 02118, USA

**Keywords:** *Orthopoxvirus*, Monkeypox virus, Type I interferon, IFN-β, MxA

## Abstract

**Background:**

The *Orthopoxvirus *genus contains numerous virus species that are capable of causing disease in humans, including variola virus (the etiological agent of smallpox), monkeypox virus, cowpox virus, and vaccinia virus (the prototypical member of the genus). Monkeypox is a zoonotic disease that is endemic in the Democratic Republic of the Congo and is characterized by systemic lesion development and prominent lymphadenopathy. Like variola virus, monkeypox virus is a high priority pathogen for therapeutic development due to its potential to cause serious disease with significant health impacts after zoonotic, accidental, or deliberate introduction into a naïve population.

**Results:**

The purpose of this study was to investigate the prophylactic and therapeutic potential of interferon-β (IFN-β) for use against monkeypox virus. We found that treatment with human IFN-β results in a significant decrease in monkeypox virus production and spread *in vitro*. IFN-β substantially inhibited monkeypox virus when introduced 6-8 h post infection, revealing its potential for use as a therapeutic. IFN-β induced the expression of the antiviral protein MxA in infected cells, and constitutive expression of MxA was shown to inhibit monkeypox virus infection.

**Conclusions:**

Our results demonstrate the successful inhibition of monkeypox virus using human IFN-β and suggest that IFN-β could potentially serve as a novel safe therapeutic for human monkeypox disease.

## Background

The *Orthopoxvirus *genus of the family *Poxviridae *contains a number of pathogens known to infect humans, including variola virus (VARV, the causative agent of smallpox), cowpox virus, camelpox virus, vaccinia virus, and monkeypox virus (MPXV). Human infection with members of this genus results in varying degrees of morbidity and mortality. Virions are enveloped and brick-shaped, with a dumbbell shaped core containing the genetic material [1]. *Orthopoxviruses *contain a single, linear piece of double-stranded DNA with highly conserved central regions and more variable terminal ends [1]. The proteins expressed from the terminal ends are predominantly involved in immunomodulation and/or host range determination [2-4].

VARV, the etiological agent of smallpox, causes an acute, systemic lesional disease with a mortality rate of approximately 30% [5,6]. Eradicated in 1977, smallpox remains a constant threat due to its potential use as a biological weapon for mass dissemination to a largely unprotected worldwide population. Unfortunately, VARV is not the only member of the *Orthopoxvirus *genus that causes severe disease in humans and has the potential for development as a biological weapon. The global eradication of smallpox and the subsequent cessation of smallpox vaccination in 1980 allowed for the emergence of another lethal zoonotic disease, monkeypox.

Similar to smallpox, monkeypox is a systemic lesional disease with a prodrome period of flu-like symptoms (fever, malaise, chills, headache) followed by the development of a progressive maculopapular rash which expands in a centrifugal pattern and progresses from papules to vesicles to pustules and finally to crusts [7-11]. MPXV is a zoonotic virus endemic in the Democratic Republic of the Congo (DRC) where it regularly emerges from reservoir species, including squirrels and other rodents [12-14], to cause serious disease outbreaks in humans. The best estimate of mortality rate is approximately 10%; however, this is likely an underrepresentation due to sporadic reporting since 1986 and a lack of information concerning the complete geographic range of human monkeypox disease [9,15-18].

There are 2 distinct clades of MPXV, West African and Central African. MPXV strains belonging to the West African clade are far less virulent than Central African strains in both humans and non-human primates, with diminished morbidity and human-to-human transmissibility [19,20]. The MPXV outbreak that occurred in the Midwestern United States in 2003 was caused by a West African strain of MPXV and thus resulted in less severe disease than what is typically seen in outbreaks in Central Africa [21]. This outbreak did, however, demonstrate the ability of MPXV to reach beyond the African continent and cause disease in MPXV-naïve populations. Although outbreaks of Central African monkeypox have not been seen outside of Africa, predictions based on an ongoing active disease surveillance study in the DRC suggest that spread to a MPXV-naïve population could have significant public health impacts. This study was conducted in nine health zones in the DRC and revealed a dramatic increase in monkeypox cases, with 760 laboratory confirmed cases identified from 2005 to 2007 [18]. Although previous vaccination against smallpox was found to still confer significant protection, only approximately 25% of the population in the sampled health zones had evidence of past vaccination [18]. Data suggesting that the incidence of human-to-human transmission of MPXV is on the rise in this region is also concerning [18,22] and could suggest that fading herd immunity coincident with a rise in the number of unvaccinated persons is allowing for more efficient spread. Additionally, it is possible that genetic variants are emerging that are more highly adapted to humans. Taken together with a long incubation period, which allows for a significant period of time during which a person is potentially contagious but asymptomatic, and its potential use as a biological weapon, it is evident that the development of therapeutic methods to treat active MPXV infections is critical.

In this paper, we investigate the potential use of interferon (IFN)-β as an anti-MPXV therapeutic. IFN-β is already US Food and Drug Administration (FDA) approved for the treatment of multiple sclerosis in four forms: Betaseron, Rebif, Avonex, and Extavia. All of these products have well-defined safety records for human use (FDA).

IFN-β is a type I IFN that plays a key role in the innate immune response by promoting the production of IFN-stimulated genes that inhibit protein synthesis, induce apoptosis, and activate macrophages and natural killer cells [23-25]. Additionally, type I IFNs enhances the adaptive immune response by upregulating major histocompatibility complex-I/II expression on the surface of antigen-presenting cells [23-25].

IFNs have been generally overlooked as anti-*Orthopoxvirus *agents due to the large number of immunomodulatory proteins expressed by viruses belonging to this genus. To date, 13 *Orthopoxvirus *proteins have been shown to have anti-IFN activity: A46, A52, K7, N1, B14, K1, M2, COP-B19, B8, H1, E3, K3, and C7 [26]. Recently, a 14th protein was identified, VARV-G1R, which binds to NF-κB and inhibits NF-κB regulated gene expression [27]. Some of these proteins have also been shown to play key roles in host range determination and virulence during vaccinia virus infection. Unfortunately, most of these proteins have not been fully characterized, and the activity of orthologs expressed by MPXV and VARV has not been extensively investigated at this time. One of the best characterized of these proteins is E3. E3 has been studied in vaccinia virus and is known to block the activation of PKR [28-30]. Although VARV contains a full length and fully functional E3L, MPXV lacks the N-terminal domain responsible for binding Z-DNA and PKR [11,30,31]. Removal of this domain results in a decreased virulence in murine models of vaccinia infection [32]. K3 is a homolog of eIF-2α that sequesters PKR thereby preventing phosphorylation of native eIF-2α by PKR [33-35]. It is a host range gene that is expressed by both VARV and vaccinia virus but not by MPXV [11,36]. C7 and K1 have also been shown to affect the cell tropism of vaccinia virus [37-40]. Although their exact functions are less well understood, it is believed that they employ a novel mechanism to antagonize IFN [37,41]. While the role of VARV-G1 in host range restriction has not been explicitly demonstrated, G1 orthologs are present in some of the most highly pathogenic *Orthopoxviruses*, including VARV and MPXV, but not in vaccinia virus, suggesting that this protein may be a key virulence factor [27].

The detailed comparative study of COP-B19 orthologs from MPXV and VARV represents the first cross-species functional analysis of any of the anti-IFN immunomodulators [42]. In this study, COP-B19 (aka B18 or IFN α/βR) from vaccinia virus was found to react very strongly with human and murine IFN-α and IFN-β. In contrast, the VARV ortholog, B17, bound to murine IFN-β very poorly but bound to human IFN-β better than vaccinia B18. Although this study didn't give as detailed of a description of the binding properties of MPXV B16, it did suggest that immunomodulatory proteins such as COP-B19 may play significant roles in host range restriction. Additionally, it showed that the analysis of *Orthopoxvirus *immunomodulatory proteins cannot be limited to vaccinia virus but must be carried out for all *Orthopoxviruses *as orthologs may function differently and/or be affected to varying degrees by the host immune response. The genetic and functional variability of the immunomodulatory proteins necessitate that prophylactic or therapeutic agents that are intended to overcome the action of these proteins be tested for efficacy with all *Orthopoxviruses *as the susceptibility of these viruses may vary significantly.

Although IFN-β has been shown to substantially diminish vaccinia virus pathogenesis *in vivo *[43,44], the susceptibility of MPXV to IFN-β is uncertain. In this study, we found that MPXV production and release were significantly reduced in the presence of IFN-β. Additionally, IFN-β was able to inhibit MPXV when introduced 6-8 h after infection, revealing its potential for use as a therapeutic against established infections. IFN-β treatment was able to induce the expression of the antiviral protein MxA during MPXV infection, and constitutive expression of MxA was able to inhibit virus production. Collectively, the data show that IFN-β is a strong novel candidate for further investigation as a prophylactic and therapeutic against MPXV.

## Results

### MPXV was inhibited by IFN-β

**Figure 1:**
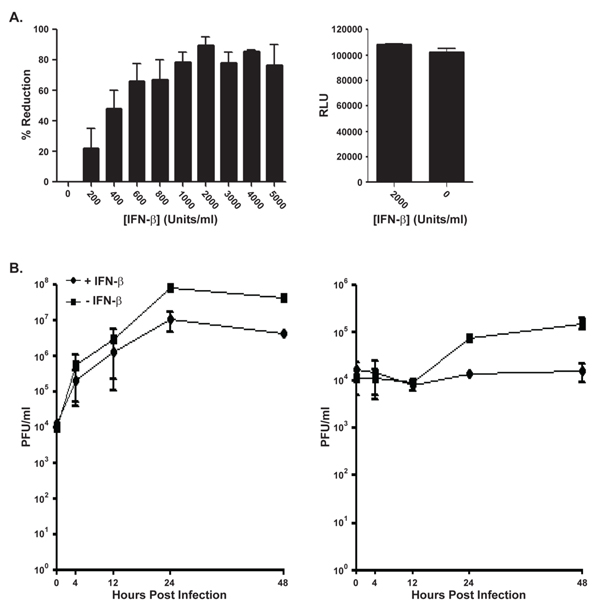
**(**a**) IFN-β titration**. HeLa cells were pre-treated for 24 h with the indicated concentrations of IFN-β. After pre-treatment, the cells were either left uninfected (right) or were infected with MPXV-Zaire at an MOI = 5 in the presence of the indicated concentrations of IFN-β (left). Infected cells were harvested 24 h p.i., and lysates were titered by plaque assay. Uninfected cells were assayed for viability using a CellTiter-Glo Luminescent Cell Viability Assay. RLU = relative light units. (**b**) High MOI growth curve. HeLa cells were either left untreated or were pre-treated for 24 h with 2000 U/ml of IFN-β. After pre-treatment, the cells were infected with MPXV-Zaire (MOI = 5) in the presence or absence of 2000 U/ml of IFN-β. Cells (left) and medium (right) were separately harvested at the indicated times, and lysates were titered by plaque assay. Note the different scales in the left and right panels.

Although IFN-β has been used alone and in combination with other cytokines in studies involving vaccinia virus, the response of MPXV to IFN-β treatment had not been previously investigated. HeLa cells that were pre-treated for 24 hours (h) before infection with increasing concentrations of IFN-β (0-5000 units [U]/ml) were infected with MPXV-Zaire at a high multiplicity of infection (MOI). The cells were harvested 24 h post infection (p.i.) and virus present titered by plaque assay. Titration results indicated that MPXV was susceptible to inhibition by IFN-β with concentrations as low as 600 U/ml, and an optimal approximately 91% reduction was seen with 2000 U/ml of IFN-β (Figure 1a). Examination of IFN-β treated HeLa cells using the CellTiter-Glo Luminescent Cell Viability Assay (Promega, Madison, WI) confirmed that there was no observable toxicity from IFN treatments (Figure 1a). Based on the dose response curves and lack of observable cellular toxicity, a 2000 U/ml 24 h pre-treatment was selected for further experimentation.

To specifically investigate infectious virus production and release in the presence of IFN-β, a high MOI growth curve using MPXV-Zaire was performed. Although virus replication still occurred in cells treated with IFN-β, we observed an approximately 1 log reduction in cell-associated and released virus 24-48 h p.i. in the presence of IFN-β compared to untreated controls (Figure 1b).

**Figure 2:**
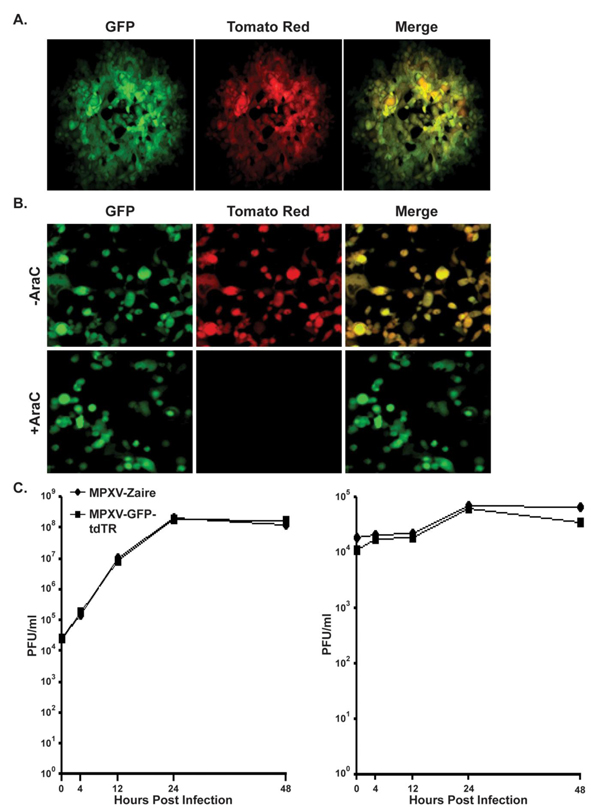
**Characterization of MPXV-GFP-tdTR**. (**a**) Vero-E6 cells infected with MPXV-GFP-tdTR were imaged 48 h p.i. by fluorescence microscopy. A single representative plaque is shown. Green is GFP, red is Tomato Red, and yellow represents the overlap of green and red fluorescence. (**b**) Vero-E6 cells infected with MPXV-GFP-tdTR (MOI = 5) in the presence or absence of Ara-C were imaged after 48 h by fluorescence microscopy. Green is GFP, red is Tomato Red, and yellow represents the overlap of green and red fluorescence. (**c**) High MOI growth curve. HeLa cells were infected with MPXV-GFP-tdTR at an MOI = 5. Cells (left) and medium (right) were separately harvested at the indicated times, and lysates were titered by plaque assay. Note the different scales for cell and media graphs.

The recombinant virus MPXV-GFP-tdTR, which expresses green fluorescent protein (GFP) from an early MPXV promoter and Tomato Red (TR) from a late MPXV promoter, allows visualization of early and late gene expression. Fluorescence microscopy of recombinant plaques demonstrated uniform distribution and complete colocalization of GFP and TR signal (Figure 2a). Cells infected in the presence of cytosine-β-D-arabinofuranoside (Ara-C), which allows early gene expression but inhibits DNA replication and subsequently late gene expression, showed a complete knockdown of only TR expression (Figure 2b), verifying the expression profile of GFP and TR. A time course experiment using flow cytometry also demonstrated GFP expression as early as 2 h p.i. and TR expression between 9 and 12 h p.i. (data not shown). Growth curves using high MOI inoculums were performed with both wild type and MPXV-GFP-tdTR and showed no significant difference in the amount of cell-or medium-associated virus (Figure 2c), indicating that incorporation of the two fluorescent genes had no impact on virus growth kinetics.

**Figure 3:**
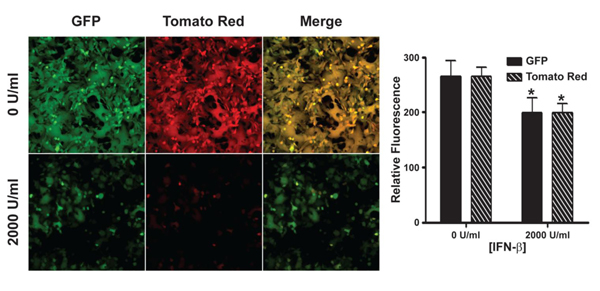
**HeLa fluorescence**. HeLa cells were pre-treated for 24 h with the indicated concentrations of IFN-β. After pre-treatment, the cells were infected with MPXV-GFP-tdTR (MOI = 5) in the presence or absence of 2000 U/ml of IFN-β. The cells were imaged 24 h p.i. by fluorescence microscopy (left), and relative fluorescence was assayed using a fluorescence microplate reader (right). Green is GFP, red is Tomato Red, and yellow represents the overlap of green and red fluorescence.

Fluorescence microscopy and a relative fluorescence assay of IFN-β pre-treated, MPXV-GFP-tdTR infected cells revealed a significant reduction of GFP expression in the presence of IFN-β compared to untreated controls (Figure 3). TR expression was also reduced compared to untreated controls (Figure 3); however, the amount of GFP and TR expression in treated cells was similar, suggesting a role for IFN-β in blocking early gene expression.

**Figure 4:**
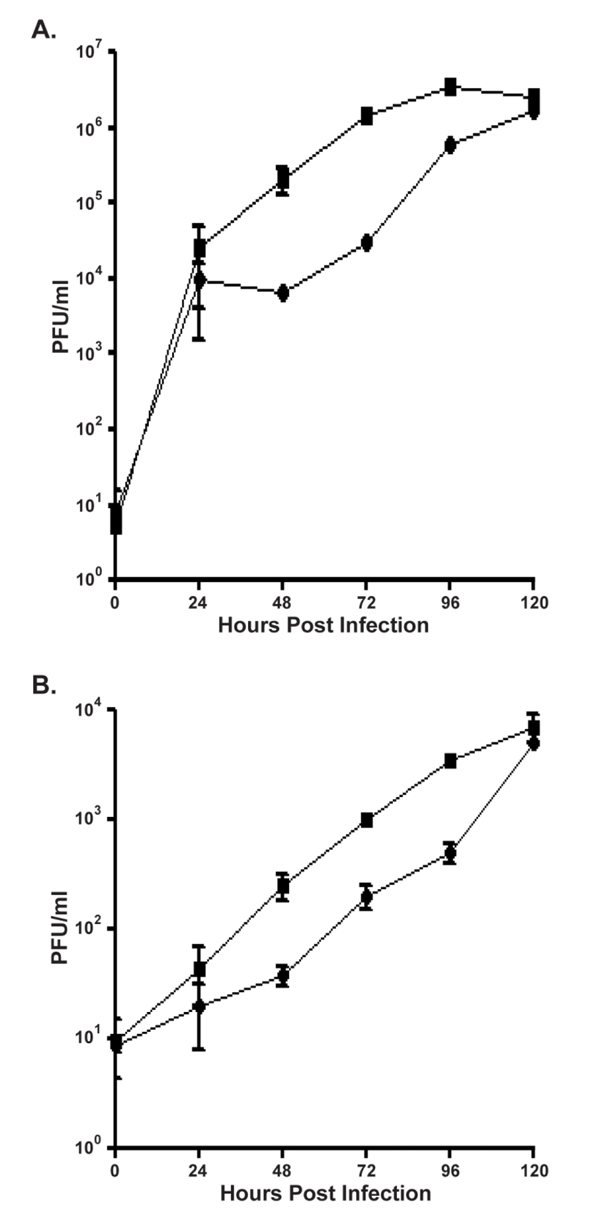
**Low MOI growth curve**. HeLa cells were either left untreated or were pre-treated for 24 h with 2000 U/ml of IFN-β. After pre-treatment, the cells were infected with MPXV- Zaire at an MOI = 0.01 in the presence or absence of 2000 U/ml of IFN-β. Cells (**a**) and medium (**b**) were separately harvested at the indicated times, and lysates were titered by plaque assay. Note the different scales in (**a**) and (**b**).

Pathogenesis in a host system requires that MPXV spread efficiently from cell to cell. To look at cell-to-cell spread *in vitro*, we performed a low MOI growth curve using MPXV-Zaire. In the presence of IFN-β, the amount of cell-associated virus was reduced by greater than a log at 48-72 h p.i. (Figure 4a); however, by 120 h p.i., infectious virus levels were comparable to untreated controls. A similar trend was observed when medium-associated virus was titered (Figure 4b). Therefore, MPXV spread is attenuated *in vitro *in the presence of IFN-β.

### Expression of IFN-induced MxA inhibits MPXV infection

**Figure 5:**
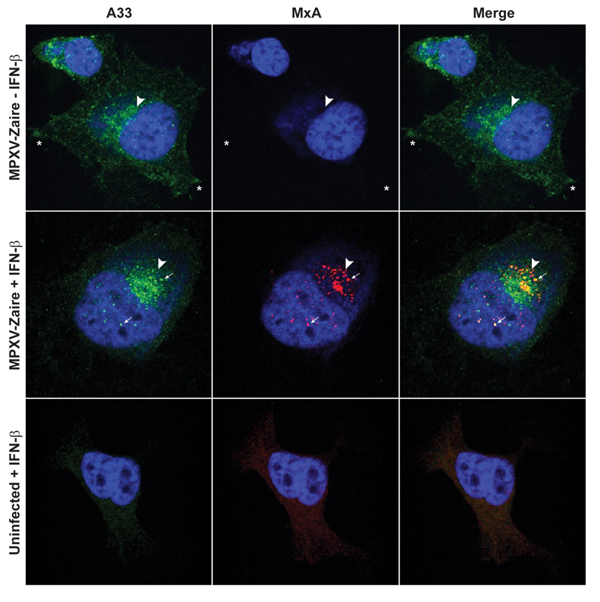
**Confocal microscopy of MxA in infected cells**. HeLa cells on coverslips were either left untreated or were pre-treated for 24 h with 2000 U/ml of IFN-β. After pre-treatment, the cells were infected with MPXV-Zaire at an MOI = 1 in the presence or absence of 2000 U/ml of IFN-β. Coverslips were harvested, fixed, permeabilized, and immunostained for MxA and A33 24 h p.i.. The coverslips were stained with Hoechst dye, mounted onto slides, and imaged by confocal microscopy. Green is A33, red is MxA, blue is Hoechst dye, and yellow represents the overlap of green and red fluorescence. Arrowheads point to the site of envelopment, arrows point to distinct areas of signal overlap, and asterisks denote the cell vertices.

MxA is a large GTPase induced by IFN that has been shown to have antiviral activity [45-51]. MxA expression in cells infected with MPXV in the presence of IFN-β was assessed. Immunostains for MxA and the viral protein A33 demonstrated a diffuse cytoplasmic pattern of MxA in uninfected, IFN-β pre-treated cells that was consistent with previous reports [52] while MxA expression in MPXV infected cells was only observed when cells were pre-treated with IFN-β (Figure 5). In infected cells treated with IFN-β, MxA was localized into distinct punctate regions, and some co-localization with A33 in the cytoplasm and at the site of wrapping was observed (Figure 5).

**Figure 6:**
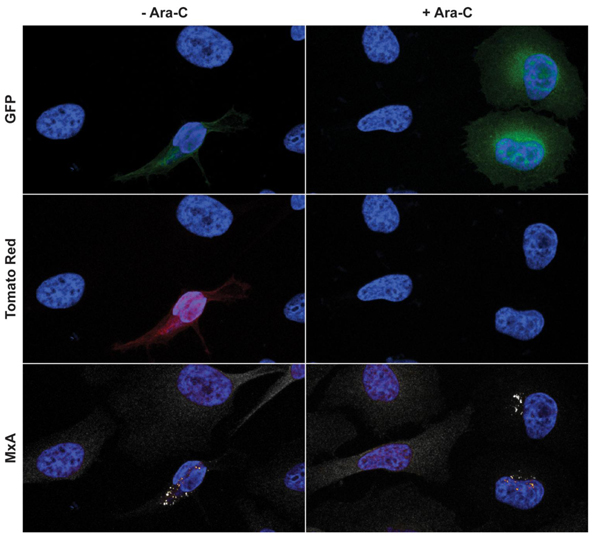
**MxA relocalization in the presence of Ara-C**. HeLa cells on coverslips were pre-treated for 24 h with 2000 U/ml of IFN-β. After pre-treatment, the cells were infected with MPXV-GFP-tdTR at an MOI = 1 in the presence 2000 U/ml of IFN-β and in the presence or absence of Ara-C. Coverslips were harvested, fixed, permeabilized, and immunostained for MxA 24 h p.i.. The coverslips were stained with Hoechst dye, mounted onto slides, and imaged by confocal microscopy. Green is GFP, red is Tomato Red, blue is Hoechst dye, and white is MxA.

To determine whether MPXV late protein production was necessary for the re-distribution of MxA, infected (and IFN-β treated) cells were incubated with Ara-C which inhibits viral replication and subsequent late gene expression. Similar punctate regions of MxA staining were observed in both the presence and absence of Ara-C in infected cells (Figure 6), demonstrating that neither replication nor late protein production is necessary for the re-localization of MxA during MPXV infection.

**Figure 7:**
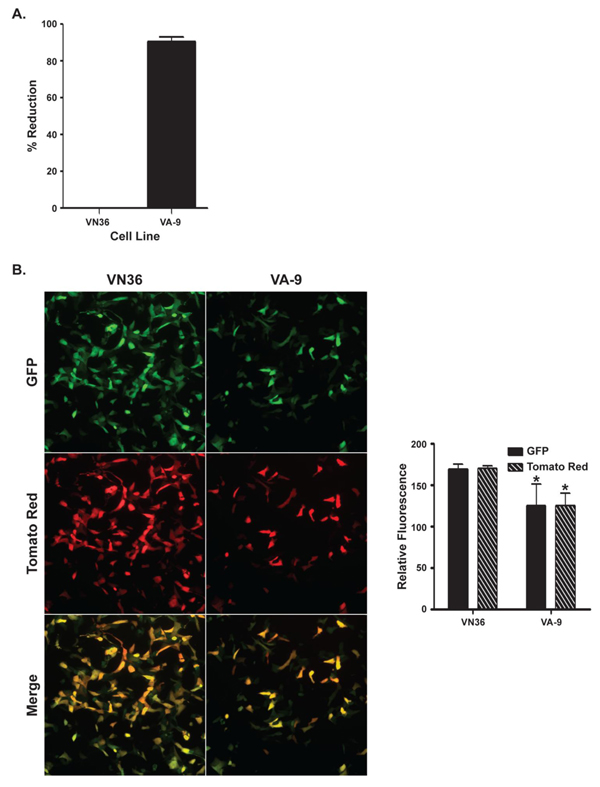
**Infection of cells constitutively expressing MxA**. (**a**) VA-9 and VN36 cells were infected with MPXV-Zaire at an MOI = 5. The cells were harvested and lysed 24 h p.i., and the amount of virus present in the lysates was titered by plaque assay. (**b**) VA-9 and VN36 cells were infected with MPXV-GFP-tdTR at an MOI = 5. The cells were imaged 24 h p.i. by fluorescence microscopy (left), and relative fluorescence was measured using a fluorescence microplate reader (right). Green is GFP, red is Tomato Red, and yellow represents the overlap of green and red fluorescence.

VA-9, a cell line that constitutively expresses MxA, [53] and the parental control cell line VN36 [53] were infected with MPXV-Zaire at a high MOI to look at the antiviral activity of MxA against MPXV. We observed an approximately 91% reduction in the amount of infectious virus present in VA-9 cells compared to VN36 control cells (Figure 7a). To confirm these results, we performed fluorescence microscopy on VA-9 and VN36 cells infected with MPXV-GFP-tdTR at a high MOI, and the relative fluorescence was measured. Again, we observed a statistically significant inhibition of infection as both early and late gene expression appeared to be equally reduced in the presence of MxA (Figure 7b), demonstrating a role for MxA in the inhibition of MPXV by IFN-β.

**Figure 8:**
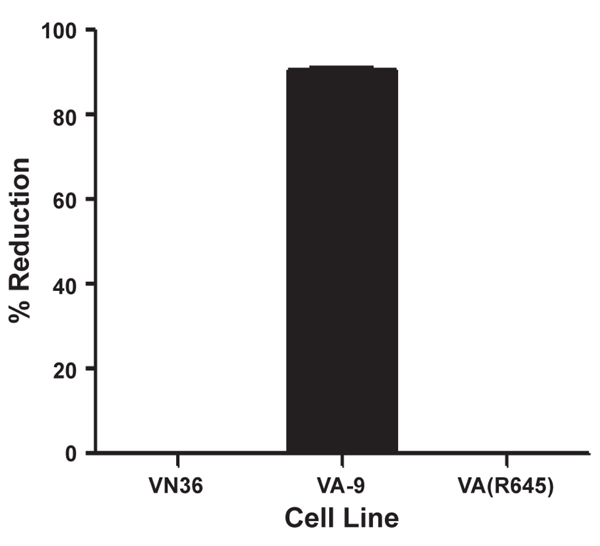
**MPXV resistance to MxA(E645R)**. VA-9, VA(R645), and VN36 cells were infected with MPXV-Zaire at an MOI = 5. The cells were harvested and lysed 24 h p.i., and the amount of virus present in the lysates was titered by plaque assay.

It was previously shown that a mutant form of MxA containing a Glu-to-Arg substitution at amino acid 645 [MxA(E645R)] lost its ability to inhibit infection by vesicular stomatitis virus and African swine fever virus (ASFV) but retained its inhibitory effect over influenza virus and thogoto virus [49,53,54]. To test the susceptibility of MPXV to this mutant MxA, VA(R645) cells were infected with MPXV-Zaire at an MOI = 5. Although we still observed an approximately 91% inhibition in VA-9 cells, we observed no reduction of infectious virus production in VA(R645) cells (Figure 8), suggesting that MPXV is resistant to inhibition by MxA(E645R).

### Post infection treatment with IFN-β was able to inhibit MPXV-Zaire

**Figure 9:**
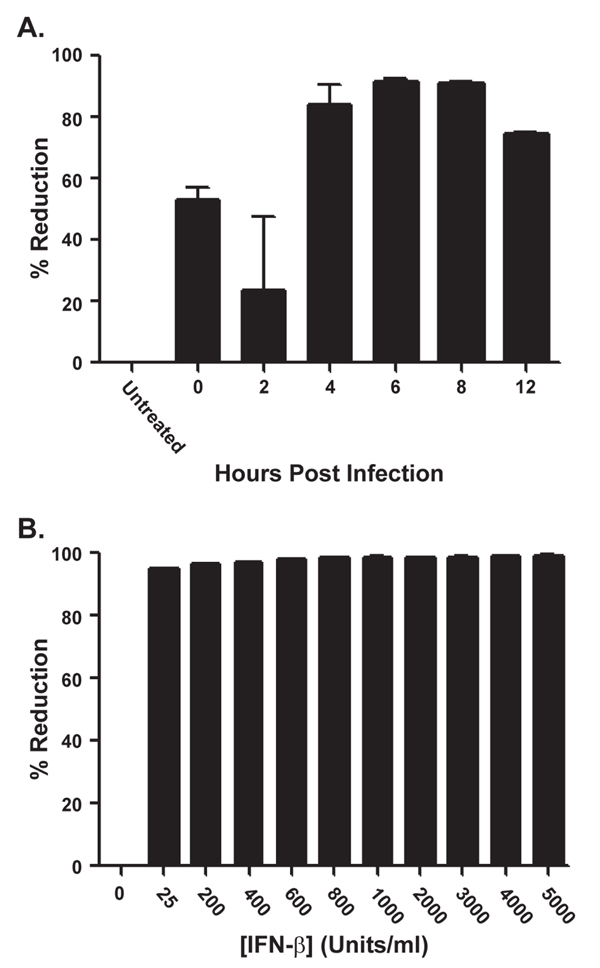
**(**a**) Post-treatment assay**. HeLa cells infected with MPXV-Zaire at an MOI = 5 in the presence or absence of 2000 U/ml of IFN-β were treated with 2000 U/ml of IFN-β at the indicated times after infection. The cells were harvested 24 h p.i. and lysates were titered by plaque assay. (**b**) Infection of fibroblast with MPXV. Normal human dermal fibroblasts were pre-treated for 24 h with 2000 U/ml of IFN-β. After pre-treatment, the cells were infected with MPXV-Zaire (MOI = 5) in the presence or absence of 2000 U/ml of IFN-β. The cells were harvested 24 h p.i., and the amount of virus present in the lysates was titered by plaque assay.

To test the therapeutic limit of IFN-β, HeLa cells infected at a high MOI with MPXV-Zaire were treated with IFN-β at 0, 2, 4, 6, 8, or 12 h p.i.. An approximately 91% reduction in infectious virus was observed when IFN-β was added 6-8 h p.i. (Figure 9a), suggesting that IFN-β can significantly inhibit MPXV when added during an active infection.

### MPXV is highly susceptible to IFN-β inhibition in human primary cells

Human primary cells more closely resemble an *in vivo *situation than immortalized cell lines (such as HeLa cells) and were, therefore, used to examine the susceptibility of MPXV under more physiologically relevant conditions. Normal human dermal fibroblasts that were pre-treated for 24 h before infection with increasing concentrations of IFN-β (0-5000 U/ml) were infected with MPXV-Zaire at a high MOI. The cells were harvested 24 h p.i. and virus present titered by plaque assay. An approximately 95% reduction in infectious virus was observed with the lowest concentration of IFN-β (25 U/ml), with maximum inhibition of approximately 99% observed at concentrations greater than or equal to 1000 U/ml (Figure 9b). In HeLa cells, this level of inhibition was not even seen with the highest concentration of IFN-β (5000 U/ml) (Figure 1a), demonstrating that MPXV susceptibility to IFN-β is enhanced when primary cells are used.

## Discussion

In this report, we described the potential use of IFN-β as a novel anti-MPXV therapeutic. Previous reports have shown that exogenously introduced type I IFN protects non-human primates from lesion development after vaccinia virus challenge [44], and IFN-β expressed by a recombinant vaccinia virus is 100% effective at preventing mortality in mice [43,44]. Here, we showed that IFN-β is capable of significantly reducing MPXV infection. Fluorescence microscopy suggested that IFN-β treatment resulted in an antiviral state that is capable of interfering with infection, and high and low MOI growth curves demonstrated that MPXV production, release, and spread were reduced by IFN-β.

Based on immunological studies of IFN-β, it is likely that the effect of IFN-β on MPXV is multifaceted and dependent on numerous effector molecules belonging to the Type I IFN signaling cascade. In this report, we showed that one such molecule, MxA, has a significant inhibitory effect on MPXV. MxA has antiviral activity against numerous RNA viruses including influenza viruses, bunyaviruses, thogoto virus, measles virus, human parainfluenza virus 3, vesicular stomatitis virus, Semliki Forest virus, and hepatitis B virus [45-51], as well as the DNA-containing ASFV [49]. Like MPXV, ASFV is a large, double stranded DNA virus that replicates entirely in the cytoplasm of the infected cell. Similar to ASFV, we showed that MPXV is inhibited by MxA but is resistant to inhibition by the mutant MxA(E645R). Additionally, MxA is relocalized following infection with both MPXV and ASFV, and this relocalization is independent of late protein production. In ASFV infected cells, MxA appears to be recruited to the site of virus assembly. Similarly, the majority of MxA in MPXV infected cells appeared to be located at the site of virus envelopment. MxA relocalization was still observed in the presence of Ara-C during MPXV infection but not ASFV infection, suggesting that MxA relocalization is independent of MPXV replication. We did not investigate the specific mode of action of MxA against MPXV as this was beyond the scope of this study which focused predominantly on the effectiveness of IFN-β against MPXV as justification for its further development as a therapeutic against highly pathogenic *Orthopoxviruses*. However, the data suggest that the method by which MxA inhibits MPXV might be similar to its inhibition of ASFV.

There is evidence in the literature that *in vitro *tests likely underestimate the inhibitory effect of IFNs on *Orthopoxviruses in vivo*, where additional immune defenses could act in concert with IFN signaling. Type I IFN has been shown to protect mice and non-human primates from morbidity and mortality after challenge with the closely related vaccinia virus [43,44]. Additionally, studies have shown that as little as a 1-2 log reduction in MPXV significantly reduces morbidity and mortality in non-human primates [55]. We, therefore, hypothesize that the effect of IFN-β on MPXV infectivity would be even greater *in vivo*, resulting in significant reductions in morbidity and mortality. In support of this prediction, we observed a much greater reduction in infectious MPXV by IFN-β when a primary cell line (normal human dermal fibroblast cells), which more closely mimics the *in vivo *state, was used. Collectively, the data presented in this report support the further development of IFN-β as a novel anti-MPXV countermeasure.

## Conclusions

Thirty years after the cessation of the smallpox vaccination campaign, dramatic increases in MPXV prevalence in the DRC [18] have raised concerns that the increasing size of the unvaccinated population is allowing for higher rates of zoonosis, and suggest a much greater risk of transmission to other susceptible populations worldwide. In 2003, a chain of seven generations of uninterrupted human-to-human spread revealed that an unprotected population could potentially sustain a MPXV outbreak [11]. Additionally, mutations that result in a virus that is better adapted to humans could strengthen transmissibility and pathogenesis. Based on information gained during active disease surveillance studies in the DRC [18], there is growing concern that the introduction of a virulent strain of MPXV into an area where little or no anti-*Orthopoxvirus *immunity exists, such as in the United States, could result in an epidemic with significant public health implications. Therefore, the development of countermeasures against MPXV and closely related VARV is of great importance.

To this day, vaccination still remains the most effective anti-*Orthopoxvirus *prophylactic. However, these vaccines are the most reactogenic of all FDA-approved vaccines, prompting the cessation of their generalized use in 1980 due to the unnecessary risk of complications in the absence of an active smallpox epidemic [56,57]. Under current guidelines these vaccines will be contraindicated for 1 in 5 individuals [5,58,59], including individuals who have heart disease, skin disorders, and/or have a weakened immune system [57]. The risks associated with live vaccines have prompted the investigation into safer alternatives, including attenuated and subunit vaccines [56,57]. The development of therapeutics that can treat established infections caused by outbreak or accidental exposure (such as by laboratory accident) or to minimize/treat adverse events caused by vaccination is ongoing, and currently no therapeutics have been licensed for widespread use against *Orthopoxviruses*. Additionally, the development of at least two therapeutics with distinct mechanisms of action is required before the destruction of the remaining stores of VARV can even be considered. Cidofovir and its derivatives [60-63] and Gleevec [64,65] have been investigated for this purpose. Presently the most promising candidate is ST-246 which specifically targets the virus by inhibiting the viral protein F13 [55,66]. In this report, we investigated the *in vitro *effectiveness of a novel candidate therapeutic, IFN-β, against MPXV. IFN-β is an attractive therapy because it is already in use to treat multiple sclerosis, it is readily available and has a well-defined safety profile, and it could be quickly implemented and used off-label. Due to the limited amount of information available concerning the function of the various anti-IFN proteins expressed by *Orthopoxviruses *(reviewed in the introduction), particularly the highly pathogenic MPXV and VARV, data obtained from investigating the therapeutic potential of IFN-β against these viruses might also prove to be pivotal in advancing our understanding of immune evasion by *Orthopoxviruses *and uncovering the elements of an effective immune response against these pathogens. This, in turn, could provide information that would be influential in guiding the development, testing, and implementation of other anti-*Orthopoxvirus *countermeasures. IFN-β should also be considered for the treatment of adverse events associated with the smallpox vaccine, particularly for those conditions where vaccinia immunoglobulin is not recommended or is believed to be of limited utility. In conclusion, IFN-β should be further developed for prophylactic and/or therapeutic use against MPXV, as well as investigated for efficacy against other highly pathogenic *Orthopoxviruses *including VARV.

## Methods

### Cells, viruses, and interferons

VA-9, VN36, and VA(R645) cell lines [53,54] were generously provided by Dr. Otto Haller (University of Freiburg, Germany). Monolayers of HeLa cells (ATCC, Manassas, VA) and normal human dermal fibroblasts (Lonza, Walkersville, MD) were maintained in Dulbecco's Modification of Eagle's Medium (Cellgro, Manassas, VA) containing 10% heat-inactivated fetal bovine serum, 1X glutamine, and 1X penicillin/streptomycin. Monolayers of Vero-E6, MA-104, VA-9, VN36, and VA(R645) cells were maintained in Minimum Essential Medium (Cellgro, Manassas, VA) containing 10% heat-inactivated fetal bovine serum, 1X glutamine, and 1X penicillin/streptomycin. Cell counts were obtained before plating to assure that equal numbers of cells were used for all infections. All infections were performed in medium containing 2.5% heat-inactivated fetal bovine serum, 1X glutamine, and 1X penicillin/streptomycin. MPXV-Zaire-1971-005 was previously described [67,68]. Human IFN-β 1a was obtained from PBL InterferonSource (Piscataway, NJ). As described on the data sheet from the manufacturer, this product was generated from cDNA isolated from human fibroblast mRNA expressed in CHO cells; it is not PEGylated. Upon arrival, the IFN was distributed to aliquots to minimize freeze-thawing, and stored at -80°C as recommended by PBL Interferon Source to maintain activity.

### MPXV-GFP-tdTR generation and characterization

MPXV-GFP-tdTR, which expresses GFP from the early viral synthetic E/L promoter and tandem dimer Tomato Red (TR) from the late viral promoter 11, was generated as follows. An expression plasmid containing both fluorescent inserts was obtained from Dr. Grant McFadden (University of Florida). Confluent monolayers of Vero-E6 cells infected with MPXV-Zaire (MOI = 1) were transfected with 2 μg of plasmid DNA in the presence of Lipofectamine 2000 (Invitrogen, Carlsbad, CA) according to the manufacturer's instructions. Cells were harvested 24 h p.i. and lysed by freeze-thawing, and the lysate was used to infect Vero-E6 cells. Plaques that fluoresced both green and red were purified a total of 4 times on Vero-E6 cells, and then amplified to high titer in MA-104 cells. The resulting purified recombinant virus, MPXV-GFP-tdTR, contained both fluorescent tags inserted into the intergenic region between J4R and J5L. Sequencing of this region confirmed proper insertion of tdTR and GFP. A plaque assay and high MOI (MOI = 5) growth curve were performed as described previously [69] on confluent monolayers of Vero-E6 cells.

To confirm proper expression of GFP and tdTR, Vero-E6 cells were infected with MPXV-GFP-tdTR (MOI = 5) in the presence or absence of 50 μg/ml of Ara-C. After a 1 h incubation, the inoculum was removed, the cells washed, and fresh medium with and without 50 μg/ml of Ara-C was added. Images were acquired 48 h p.i. using a Nikon Eclipse te2000-s fluorescence microscope equipped with a SPOT RT Monochrome camera and overlaid using Adobe PhotoShop software.

### IFN-β titration assay

Monolayers of HeLa cells (1 × 10^6 ^cells/well) or normal human dermal fibroblasts (3 × 10^5 ^cells/well) were either left untreated or were pretreated with 0, 200, 400, 600, 800, 1000, 2000, 3000, 4000, or 5000 U/ml of human IFN-β 1a (PBL InterferonSource, Piscataway, NJ). The cells were infected 24 h later with MPXV-Zaire at an MOI of 5 in the presence or absence of IFN-β. After 1 h, the inoculum was removed, the cells washed, and fresh media with or without IFN-β was added. The cells were harvested 24 h p.i., lysed by 3 cycles of freeze-thawing/sonication, and virus titers determined by plaque assay as described above.

To assess the therapeutic limit of IFN-β, cells were treated with 2000 U/ml of IFN-β either 0, 2, 4, 6, 8, or 12 h p.i. and infections and viral titers were performed as described above.

### Growth curves

High MOI (MOI = 5) and low MOI (MOI = 0.01) growth curves were performed in duplicate as described previously [69]. Briefly, HeLa cells were either left untreated or were pre-treated with 2000 U/ml of IFN-β. The cells were infected 24 h later with MPXV-Zaire in the presence or absence of 2000 U/ml of IFN-β. For the 0 h time point, the inoculum was immediately removed, the cells were washed, 1 ml of fresh medium was added to the cells, and the cells and medium were immediately harvested. For all other time points, the inoculum was removed 1 h p.i., the cells were washed, and 1 ml of fresh medium with or without 2000 U/ml of IFN-β was added. Fresh IFN-β was added to the medium every 24 h p.i.. Cells and medium were harvested separately at 0, 4, 12, 24, and 48 h p.i. for the high MOI growth curve, and at 0, 24, 48, 72, 96, and 120 h p.i. for the low MOI growth curve. Cells were lysed by three cycles of freeze-thawing/sonication (medium harvests were not freeze-thawed to maintain the integrity of viral membranes), and viral titers were determined by plaque assay as described above.

### Fluorescence and confocal microscopy

HeLa cells grown on coverslips were either left untreated or were pre-treated 24 h before infection with 2000 U/ml of IFN-β. Cells were infected 24 h later with MPXV-GFP-tdTR or MPXV-Zaire at a MOI of 5 in the presence or absence of IFN-β and/or Ara-C (50 μg/ml). Cells were either imaged 24 h p.i. by using aNikon Eclipse te2000-s fluorescence microscope equipped with a SPOT™ RT Monochrome camera (and overlaid using Adobe PhotoShop software) or were fixed in phosphate-buffered saline (PBS) containing 4% paraformaldehyde in preparation for immunostaining. Fixed cells were permeabilized with PBS containing 0.1% Triton X-100 (Sigma-Aldrich, St. Louis, MO), quenched with PBS containing 0.1X glycine (Sigma-Aldrich, St. Louis, MO), and stained with an anti-MxA mAb (Sigma Aldrich, St. Louis, MO) followed by either Alexa Fluor 568-conjugated goat anti-rabbit IgG (Invitrogen, Carlsbad, CA) or Alexa Fluor 647-conjugated goat anti-rabbit IgG (Invitrogen, Carlsbad, CA). Additionally, cells infected with MPXV-Zaire were stained with an anti-A33 monoclonal antibody (mAb) (generously provided by Dr. Jay Hooper, USAMRIID) followed by Alexa Fluor 488-conjugated goat anti-mouse IgG (Invitrogen, Carlsbad, CA). The coverslips were thoroughly washed and, following staining with Hoechst dye for 30 min, were mounted onto slides using Fluoromount-G (SouthernBiotech, Birmingham, AL). Images were captured using a Leica TCS SP5 confocal microscope and overlaid using Leica Application Suite software. All relative fluorescence measurements were acquired using a SpectraMax Gemini EM Fluorescence Microplate Reader (Molecular Devices, Sunnyvale, CA).

### Infection of VA-9, VN36, and VA(R645) cell lines

VA-9, VN36, and VA(R645) cells were infected with MPXV-Zaire or MPXV-GFP-tdTR at an MOI of 5. After absorption for 1 h, the inoculum was removed, the cells were washed, and fresh medium was added. The cells infected with MPXV-Zaire were harvested 24 h p.i. and lysed by three cycles of freeze-thawing/sonication, and viral titers were determined by plaque assay as described above. Fluorescence microscopy was performed 24 h p.i. on cells infected with MPXV-GFP-tdTR using a Nikon Eclipse te2000-s fluorescence microscope equipped with a SPOT RT Monochrome camera and overlaid using Adobe PhotoShop software. Relative fluorescence measurements were acquired using a SpectraMax Gemini EM Fluorescence Microplate Reader (Molecular Devices, Sunnyvale, CA).

## Abbreviations

IFN: Interferon; VARV: Variola virus; MPXV: Monkeypox virus; DRC: Democratic Republic of Congo; FDA: US Food and Drug Administration; H: Hours; PI: Post infection; Ara-C: Cytosine-β-D-arabinofuranoside; MOI: Multiplicity of infection; GFP: Green fluorescent protein; tdTR: Tandem dimer Tomato Red; ASFV: African swine fever virus; U: Units; PBS: Phosphate-buffered saline.

## Competing interests

The authors declare that they have no competing interests.

## Authors' contributions

SCJ designed the study, conducted the majority of the assays presented, and drafted the manuscript. KLL performed the generation and characterization of MPXV-GFP-tdTR. JHC provided technical advice and helped to draft the manuscript. GR performed the imaging for the confocal microscopy assays. AG provided technical support and assisted with the generation of MPXV-GFP-tdTR. LEH provided technical support and helped to draft the manuscript. All authors read and approved the final manuscript.
